# Effects of Phytoestrogen Supplement on Quality of Life of Postmenopausal Women: A Systematic Review and Meta-Analysis of Randomized Controlled Trials

**DOI:** 10.1155/2019/3261280

**Published:** 2019-04-01

**Authors:** Ching-Ching Peng, Chia-Yu Liu, Nai-Rong Kuo, Tao-Hsin Tung

**Affiliations:** ^1^Department of Community and Family Medicine, Cheng Hsin General Hospital, Taipei, Taiwan; ^2^Department of Medicine, School of Medicine, Fu-Jen Catholic University, Taiwan; ^3^Department of Medical Research and Education, Cheng Hsin General Hospital, Taipei, Taiwan; ^4^Maoming People's Hospital, Guangdong Province, China

## Abstract

**Background:**

The effect of phytoestrogen on postmenopausal quality of life is unclear. This study evaluated the effects of phytoestrogen supplement on quality of life for postmenopausal women.

**Methods:**

We conducted a systematic review and meta-analysis of randomized controlled trials on the effects of phytoestrogen supplements on the quality of life of postmenopausal women. We searched PubMed, MEDLINE, Embase, and the Cochrane Central Register of Controlled Trials on March 31, 2018, for relevant randomized controlled trials. Two authors independently selected studies, assessed risk of bias, and extracted data. Disagreement was resolved through discussion with a third author.

**Results:**

We involved 10 articles in the systematic review. 8 studies and a total of 1,129 subjects were included in the meta-analysis. The questionnaires used in the evaluation of quality of life were as follows: SF-36, 4 studies; MENQOL, 4 studies; For Short Form 36 surveys, phytoestrogen groups scored significantly higher for body pain (mean difference = 3.85, 95% confidence interval [CI] = [1.14, 6.57],* P* < 0.01), mental health (mean difference = 4.01, 95% CI = [1.49, 6.57],* P* < 0.01), and role limitations caused by emotional problems domains (mean difference = 3.83, 95% CI = [1.81, 5.85],* P* < 0.01). No statistically significant difference was obtained from Menopause-Specific Quality of Life surveys (vasomotor domain mean difference 0.14, 95% CI = [−0.08, 0.36],* P* = 0.20; physical domain mean difference 0.20, 95% CI [−0.08, 0.48],* P *= 0.15; psychological domain mean difference −0.10, 95% CI [−0.26, 0.07],* P* = 0.27; sexual domain mean difference −0.17, 95% CI [−0.42, 0.09],* P* = 0.19).

**Conclusion:**

Current evidence does not support phytoestrogen supplementation improving postmenopausal quality of life. Further comprehensive trials or long-term follow-up studies are warranted.

## 1. Introduction

Quality of life (QoL) for postmenopausal women has been a popular topic in the 21st century. Because of cessation of ovulation followed by sharp decrease in estradiol production and physiological changes, postmenopausal women may experience a degree of discomfort that is known as postmenopausal syndrome. Based on such manifestations, several scales have been developed to measure QoL for improving happiness and benefits for women after menopause. The contents of these scales generally include physical and psychological aspects. Complaints of physical problems may include hot flashes [1], vasomotor symptoms [2], pain [3], and psychological uneasiness manifesting as depression [4], anxiety, and cognitive dysfunction. Physicians may prescribe hormone therapy for these patients. Nevertheless, one study reported severe adverse events [5]. Major adverse events include coronary heart disease, breast cancer, and stroke. These serious health conditions may result in a significant reduction in QoL for patients adopting this type of treatment.

Phytoestrogen supplements are regarded as an alternative to hormone replacement therapy. This type of compound is derived mostly from plants and has structures resembling estrogen produced by the human endocrine system [6]. By binding to estrogen receptors, patients who consume such a supplement can compensate for their paucity of estrogen. Phytoestrogens are considered capable of alleviating postmenopausal symptoms. Studies have indicated the biological benefits, such as prevention of osteoporosis and cardiovascular diseases [6], of phytoestrogen on postmenopausal women. However, this remedy is likely to cause side effects, including impacts on reproductive health. Breast cancer risk, for instance, is likely to increase in patients consuming phytoestrogen supplements.

Although the consumption of phytoestrogen demonstrates physiological improvements, its effects on QoL for women after menopause remain unclear. Studies have conducted several randomized controlled trials (RCTs), but the outcomes have been inconsistent. Therefore, a more substantial and integrative result is required. The present study evaluated the effects of phytoestrogen supplements on QoL for postmenopausal women.

## 2. Materials and Methods

### 2.1. Literature Search

We conducted a systematic review and meta-analysis to assess the effect of phytoestrogen supplements on the QoL of postmenopausal women. Two researchers (Liu and Tung) searched PubMed, MEDLINE, Embase, and Cochrane Central Register of Controlled Trials from inception through March 31, 2018, for relevant publications. We did not impose any limitations on language. The search strategy is detailed in Table 1. The study protocol was registered on the PROSPERO.

### 2.2. Study Selection

Included studies met the following inclusion criteria: the study design was an RCT, participants were human, experimental group received phytoestrogen supplements, and control group received a placebo. The titles and abstracts of all studies identified by our search were independently assessed by two of the authors for eligibility. These authors checked the full text of potentially eligible trials to determine whether they met the inclusion criteria. A third author arbitrated when the two authors disagreed on inclusion of a study.

### 2.3. Data Extraction and Assessment of Potential Bias

Peng and Tung performed the data extraction and risk of bias assessment process. For all articles included, the following characteristics were obtained: first author, year, country, participants in the RCTs, characteristics of intervention, comparison groups, and outcome measurements. Because we expected different questionnaires or scales were utilized to determine QoL, we performed the meta-analyses based on the inquiry forms used.

The authors reviewed the titles and abstracts when searching the relevant studies after all references had been imported to EndNote. After a thorough appraisal of these publications, we indexed the full texts and subsequently assessed the risk of bias using the Cochrane Handbook for Systematic Reviews of Interventions [7]. The handbook includes seven domains of bias risk: (1) random sequence generation, (2) allocation concealment, (3) blinding of participants and personnel, (4) blinding of outcome assessment, (5) incomplete outcome data, (6) selective reporting, and (7) other sources of bias. Two authors independently used the Cochrane Collaboration Tool for assessing the risk of bias in the included trials [8]. Any disagreement was resolved through discussion with a third author (Peng).

### 2.4. Questionnaires Used

A comprehensive screening was conducted to identify the questionnaires in the included trials. Among all studies, the Short Form 36 (SF-36) [9] and Menopause-Specific Quality of Life (MENQOL) [10] questionnaires were the primary inquiry forms utilized to assess QoL. Other measurements included the Cervantes Health-Related Quality of Life Scale [11] and Greene Climacteric Scale [12].

The SF-36 is a widely adopted scale worldwide for evaluating patients' perception of their personal health status. It comprises the following sections to evaluate QoL: (1) vitality, (2) physical functioning, (3) body pain, (4) general health perceptions, (5) physical role functioning, (6) emotional role functioning, (7) social role functioning, and (8) mental health. These eight domains can also be categorized into two summary groups (physical and mental component summaries). After calculation according to the SF-36 Health Survey Manual and Interpretation Guide, raw scores were converted into a final scale from 0–100 that represented the overall QoL of the women.

The MENQOL is a QoL measurement introduced in 1996 and designed specifically for postmenopausal women. It is a Likert scale form that comprises 29 items that weigh the four domains of QoL for women after menopause: (1) vasomotor (items 1–3), (2) psychosocial (items 4–10), (3) physical (items 11–26), and (4) sexual (items 27–29) domains. Higher scores within a domain represent lower QoL in the respective aspect. The advantage of this widely used format is that it addresses the common postmenopausal symptoms [10]. This may yield a more dependable result for postmenopausal QoL compared with other general questionnaires. However, many medical professionals are concerned about the validity and reliability of the MENQOL for breast cancer survivors [13].

### 2.5. Statistical Analysis

We used Review Manager version 5.3.5 to calculate the overall effect of phytoestrogen supplements on QoL [14] for postmenopausal women. Heterogeneity in meta-analysis refers to variation in study outcomes between studies. In this study, we used the* χ*^2^ and *I*^2^ inconsistency statistics. The *I*^2^ statistic describes the percentage of variation across studies that is caused by heterogeneity rather than chance [15]; *I*^2^ values of 0%–24.9%, 25%–49.9%, 50%–74%, and 75%–100% were considered no, low, moderate, and high heterogeneity, respectively. A 95% confidence interval (CI) for *I*^2^ was constructed through the iterative noncentral chi-square distribution method [16]. Additionally, we applied a fixed-effect model when *I*^2^ was less than 50% and would have applied the random-effect model if *I*^2^ had been 50% or more.* P* < 0.05 indicated a significant difference between the phytoestrogen and control groups.

## 3. Results

**Figure 1:**
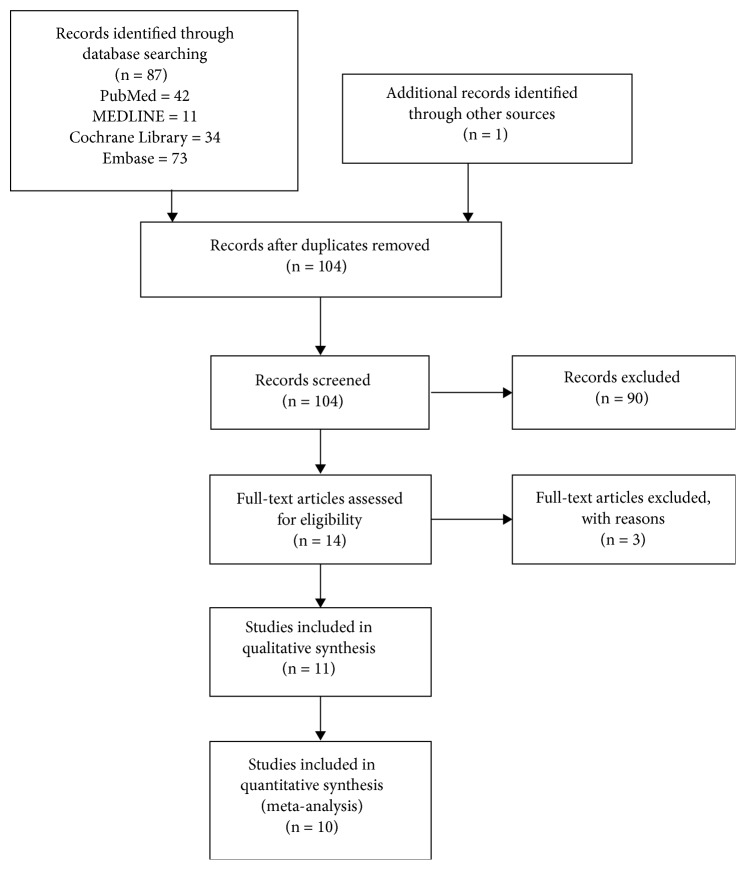
PRISMA flow diagram.

The Preferred Reporting Items for Systematic Reviews and Meta-Analyses flowchart in Figure 1 illustrates the details of our search and study selection. After a full search of the databases, we identified studies for thorough screening. Finally, ten articles were included in the systematic review and meta-analysis [17–26]. The characteristics of included studies are illustrated in Table 2. A total of 968 participants were included in the intervention group and 716 in the controls.

### 3.1. Risk of Bias Assessment

**Figure 2:**
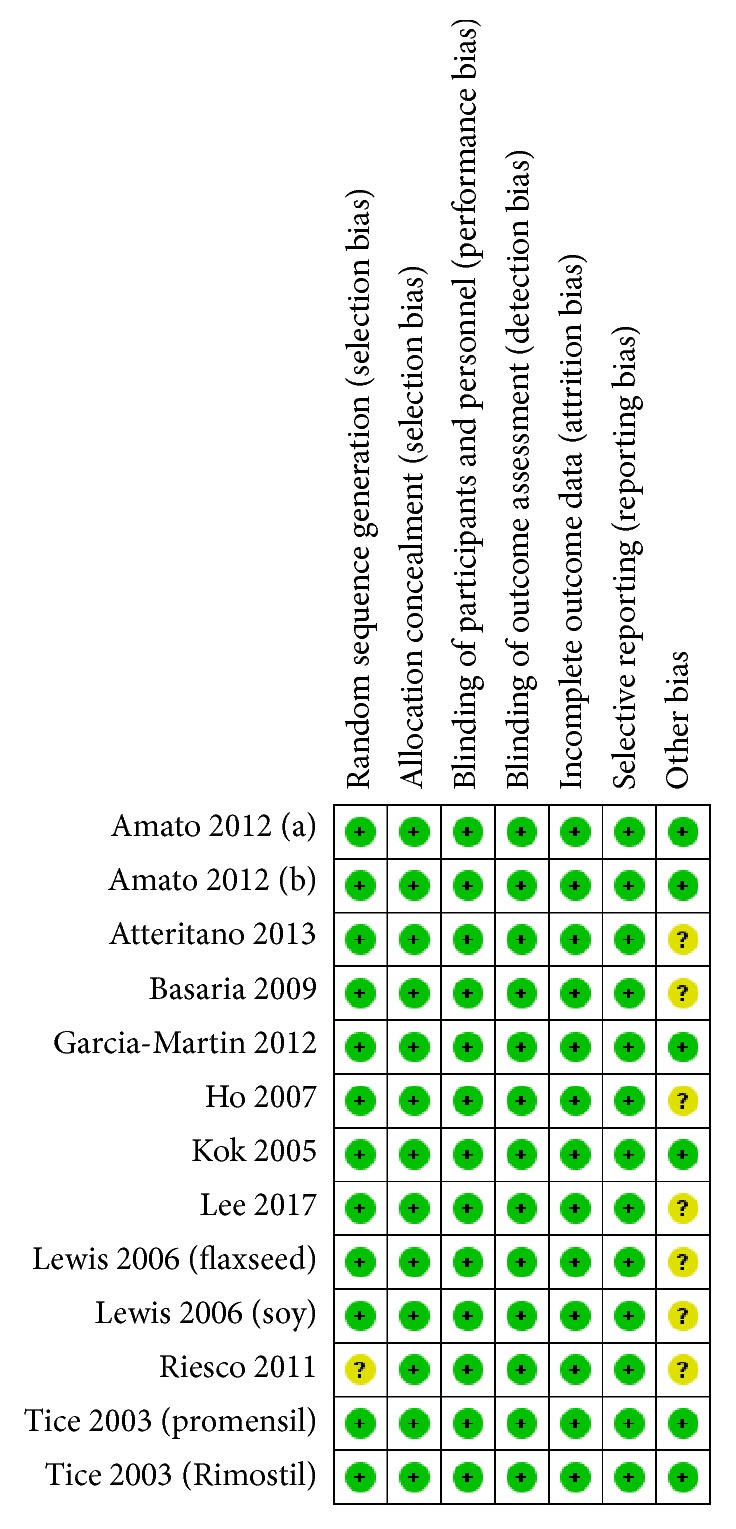
Risk of bias for included studies.

We used the Cochrane Handbook for Systematic Reviews of Interventions to assess the risk of bias in the selected studies. The result is shown in Figure 2. The overall risk of bias was low. Among all included trials, no article was assessed as high risk. One study [25] was rated an uncertain risk in random sequence generation because we could not locate the related statement in the article. We ranked seven studies [18, 19, 21, 23–25] in the “other bias” section as uncertain risks because either the confirmation of supplement consumption was unreliable (i.e., counting instead of blood sample test) or the intervention was not solely phytoestrogen.

### 3.2. Meta-Analysis and Outcome Assessment

We separately analyzed each domain of these two questionnaires and obtained a total of 12 outcomes (8 from the SF-36 [9] and 4 from the MENQOL [10]) that represented each aspect of QoL. We used Review Manager 5.3 [14] to detect statistically significant differences between the phytoestrogen and control groups.

### 3.3. QoL from SF-36 Measurement

**Figure 3:**
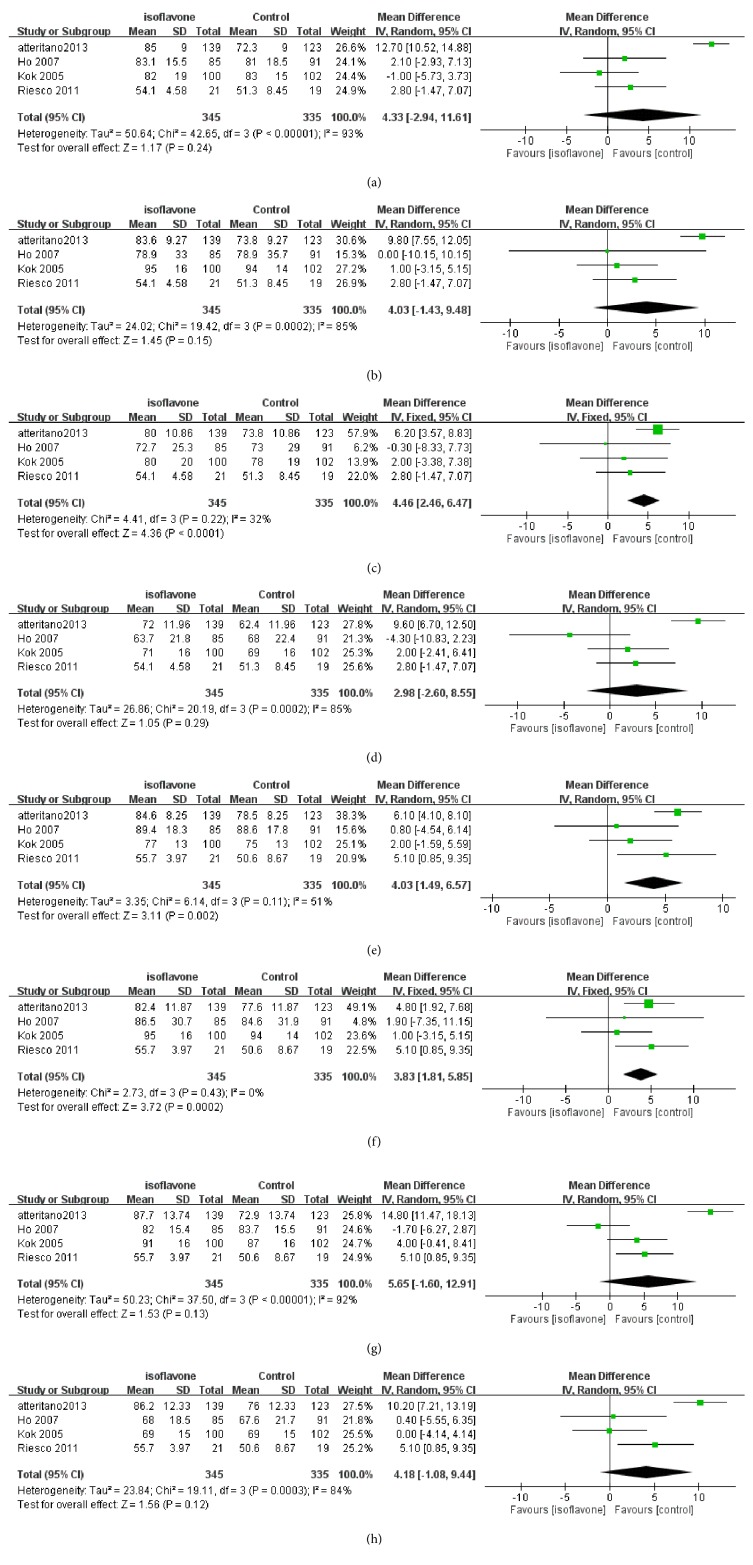
(a) Meta-analysis result of SF-36 of physical function domain. (b) Meta-analysis result of SF-36 of role limitations caused by physical problems domain. (c) Meta-analysis result of SF-36 of pain domain. (d) Meta-analysis result of SF-36 of vitality domain. (e) Meta-analysis result of SF-36 of mental health domain. (f) Meta-analysis result of SF-36 of role limitations caused by emotional problems domain. (g) Meta-analysis result of SF-36 of social function domain. (h) Meta-analysis result of SF-36 of general health perception.

Figures 3(a)–3(h) show the results of the SF-36 meta-analysis. A total of 660 subjects were involved in these subgroups of meta-analysis (329 in the case group and 331 in the controls, respectively). Three domains of the phytoestrogen group had significantly higher QoL scores compared with controls: body pain (mean score difference = 3.85, 95% CI = [1.14, 6.57]), mental health (mean score difference = 4.01; 95% CI = [1.49, 6.57]), and role limitations caused by emotional problems (mean score difference = 3.83; 95% CI = [1.81, 5.85]).

### 3.4. QoL Score from MENQOL Measurement

**Figure 4:**
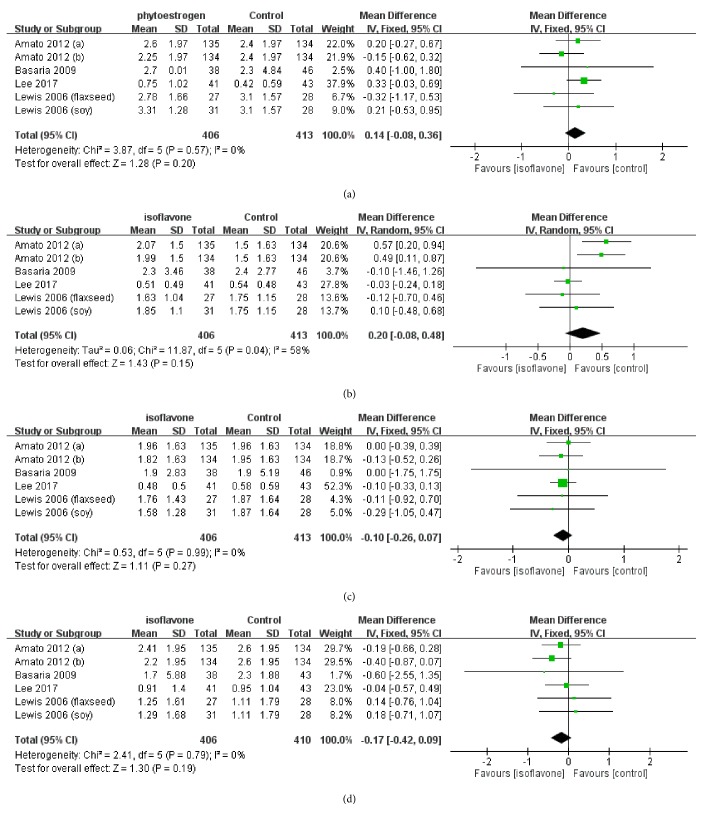
(a) Meta-analysis result of MENQOL of vasomotor domain. (b) Meta-analysis result of MENQOL of physical domain. (c) Meta-analysis result of MENQOL of psychological domain. (d) Meta-analysis result of MENQOL of sexual domain.

The MENQOL results are illustrated in Figures 4(a)–4(d). 659 individuals were included in the meta-analysis of these subgroup outcomes (408 in intervention group and 251 in the controls, respectively). No statistically significant result was obtained. The pooled QoL score differences of MENQOL domains were as follows: vasomotor domain means score difference = 0.14, 95% CI = [−0.08, 0.36]; physical domain means score difference = 0.20, 95% CI = [−0.08, 0.48]; psychological domain means score difference = −0.10, 95% CI = [−0.26, 0.07]; and sexual domain means score difference = −0.17, 95% CI = [−0.42, 0.09].

### 3.5. Publication Bias

**Figure 5:**
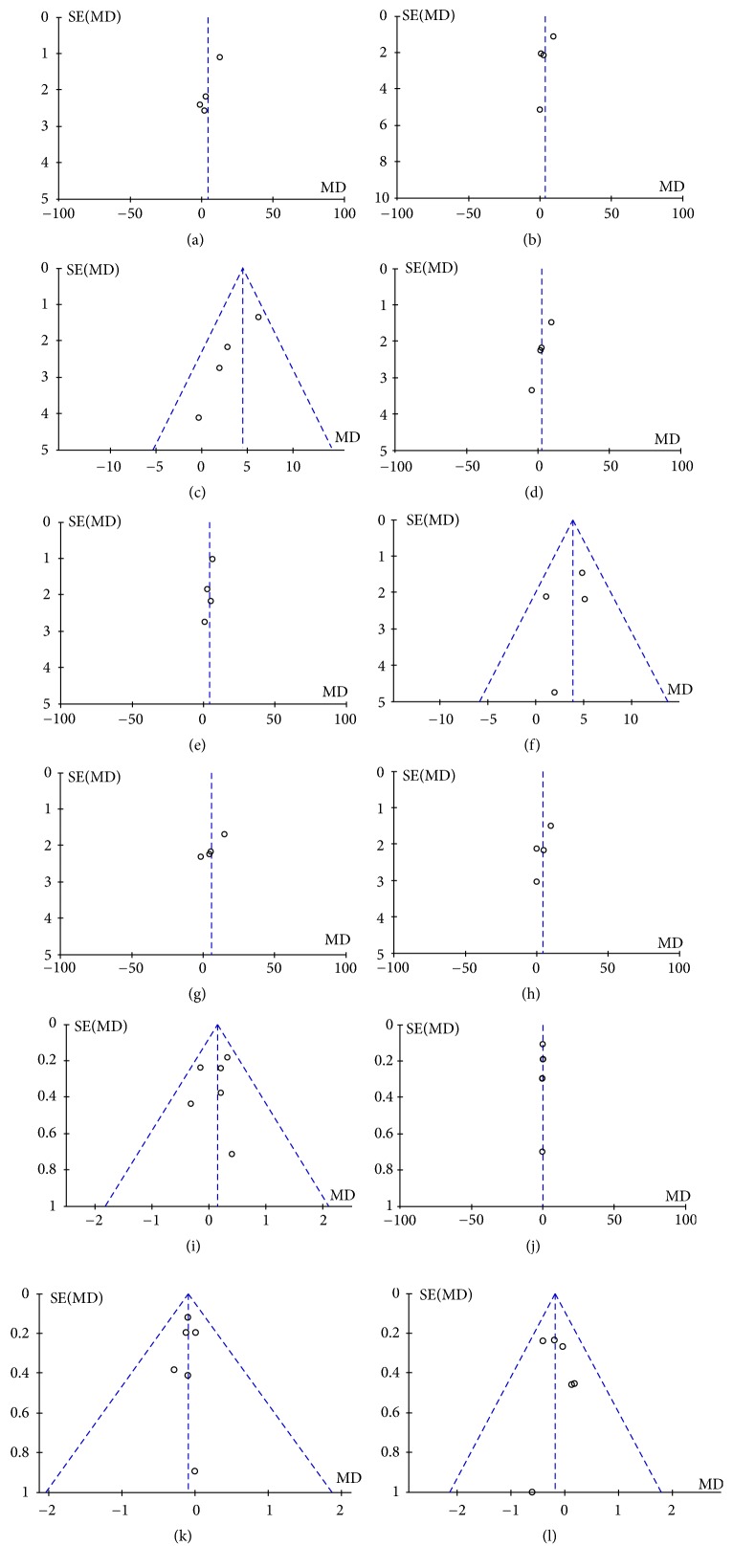
(a) Funnel plot based on SF-36 of physical function domain. (b) Funnel plot based on SF-36 of role limitations caused by physical. (c) Funnel plot based on SF-36 of pain domain. (d) Funnel plot based on SF-36 of vitality domain. (e) Funnel plot based on SF-36 of mental health domain. (f) Funnel plot based on SF-36 of role limitations caused by emotional problems domain. (g) Funnel plot based on SF-36 of social function domain. (h) Funnel plot based on SF-36 of general health perception. (i) Funnel plot based on MENQOL of vasomotor domain. (j) Funnel plot based on MENQOL of physical domain. (k) Funnel plot based on MENQOL of psychological domain. (l) Funnel plot based on MENQOL of sexual domain.

Publication bias was defined as the publication or nonpublication of studies depending on the direction and statistical significance of the results and the first systematic investigations of publication bias focused on this aspect of the problem. As Figures 5(a)–5(l) show, the funnel plot was symmetry, indicating no series publication bias in this study.

## 4. Discussion

To our knowledge, this is the first meta-analysis of QoL related to the use of phytoestrogen supplements. The exact effect of phytoestrogen on QoL remains unclear. We performed a meta-analysis and acquired inconsistent results. We concluded several reasons for these results: type of questionnaire, difference between physical and mental conditions, and characteristics of involved patients.

Outcomes after meta-analysis were inconsistent; however, results did not confirm that these supplements improve QoL. The beneficial effects on biological functions have been proven by previous studies. Nevertheless, our research did not find a robust correlation between administration of supplements and enhancement of QoL.

Many of the included studies were conducted in Western countries. Indeed, postmenopausal life has been the subject of much research recently in Western countries. Although Asian women undoubtedly experience postmenopausal symptoms, certain aspects of postmenopausal syndrome, such as vasomotor symptoms, may be more common among Caucasian women [27]. A more thorough understanding of the effects of phytoestrogen has been established in Western countries than has been in Asia because of the more diverse ethnicities, socioeconomic levels, and education levels in the Asia region.

### 4.1. Limitations

There were several methodological limitations to this study. The major limitation was the number of available RCTs was insufficient; thus, the statistical power was low because of small study sample sizes. Another limitation to this study was the controversy surrounding random-effect models; that is, the assumption of normally distributed random effects violates the basic principle of randomization in statistical inference [28]. The hypothetical common variance of these so-called random effects would serve only as a nuisance variable if there were no random effects. The end result of the application of this nuisance variable to meta-analytic weights would then be markedly increasing estimator variance and equalizing the weights through penalizing larger studies [29, 30]. Finally, the serious bias in this meta-analysis was publication bias, in the overall meta-analysis of randomized controlled trials there was no significant on the publication bias. However, it is difficult to conduct meta-regression for investigating the sources of the heterogeneity by low statistical power of insufficient studies. Further rigorous studies should be conducted with more objective measures.

### 4.2. Conclusion

In conclusion, there is a lack of solid evidence supporting the routine use of phytoestrogen supplements for improving postmenopausal QoL. Future trials should employ an adequate sample size to provide data on various subgroups, for example, different age groups. Participants of various ethnic groups should be enrolled. Both efficacy and safety outcomes should be measured and reported. Additionally, trials that compare the effects of various durations of phytoestrogen supplementation are also required to determine the optimal duration of application. Trials including a third arm with no application at all (as in the present clinical practice) are warranted to estimate the placebo effect, which might affect interpretation of the effects of phytoestrogen supplements.

## Figures and Tables

**Table 1:** Search strategy.

Database	Searching keywords
Cochrane library	(1) Postmenopause: 7873 (2) Phytoestrogen: 210 (3) Isofalvone: 665 (4) Quality of life: 74410 (5) #2 or #3: 817 (6) #1 and #4 and #5: 34

Medline	(1) exp postmenopause: 22329 (2) exp phytoestrogens: 9023 (3) exp isoflavones: 16593 (4) exp quality of life: 157718 (5) 2 or 3: 17634 (6) 1 and 4 and 5: 11

PubMed	(1) Postmenopause: 42081 (2) Phytoestrogen: 11294 (3) Isofalvone: 18640 (4) Quality of life: 320926 (5) #2 or #3: 20967 (6) #1 and #4 and #5: 42

Embase	'postmenopause'/exp AND ('phytoestrogen'/exp OR 'phytoestrogen' OR 'phytoestrogens' OR 'phytooestrogen' OR 'phytooestrogens' OR 'isoflavone derivative'/exp OR '5 hydroxy 7 prenyloxyisoflavone' OR '5 hydroxy 7, 4' dimethoxyisoflavone' OR 'isoflavone derivative' OR 'isoflavones' OR 'novasoy' OR 'novasoy 400' OR 'promensil' OR 'rimostil') AND 'quality of life'/exp; result=73

**Table 2:** Characteristics of included studies.

Author, year, country	Participants	Intervention	Comparison	Duration	Outcomes
Amato et al., 2012, U.S.	403 postmenopausal women (120 mg/day group: n=134; 80 mg/day group: n=135; placebo group: n=134)	Aglycone soy isoflavone 80 mg; aglycone soy isoflavone 120 mg	Placebo contains cellulose	2 years	MENQOL questionnaire

Attreitano et al. 2013, Italy	262 postmenopausal women with diagnosed osteopenia	Genistein (n=139)	Identical appearance placebo (n=123)	2 years	SF-36, ZSDS

Basaria et al. 2009, U.S.	84 postmenopausal women (mostly Caucasian)	Genistein 64 mg, daidzein 63 mg, glycitein 34 mg (In the form of powder mixed with beverages) (n=38)	20g of milk protein and other nutrients (same as experimental group) (n=46)	12 weeks	MENQOL questionnaire

Garcia-Martin et al., 2012, Spain	94 postmenopausal women	Soy isoflavone 50 mg/day (n=45)	Milk protein supplement (n=49)	1 year	Cervantes Health-Related Quality of Life scale

Ho et al., 2007, China	176 postmenopausal women with Chinese ethnicity that live in Hong Kong	80 mg soy derived isoflavone per day (n=91)	Identical appearance placebo (starch) (n=85)	6 months	SF-36, MMSE

Kok et al., 2005, U.S.	202 postmenopausal women	25.6 g soy protein (52 mg genistein, 41 mg daidzein, 6 mg glycitein) (n=100)	Milk protein (placebo) (n=102)	1 year	SF-36, QLS, GDS scale

Lee et al., 2017, Korea	84 postmenopausal women (n=84)	336 mg soy extract (10.5% isoflavone) and various agents (n=41)	Dextrin instead of isoflavones (n=43)	12 weeks	MENQOL questionnaire, Kupperman index

Lewis et al., 2006, Canada	87 postmenopausal women	25 g of flaxseed (50 mg lignans, n=28); 25 g of soy (42 mg of isoflavones) per day (n=31)	Wheat (for achieving similar fiber content) (n=28)	16 weeks	MENQOL questionnaire, daily hot flash frequency estimation by a 7-point Likert scale

Riesco et al., 2011, Canada	40 postmenopausal women	4 phytoestrogen capsules per day (each contain 17.5 mg of isoflavones) (n=19)	Placebo contained cellulose (n=21)	6 months	SF-36, Perceived stress scale, Kupperman index

Tice et al., 2003, U.S.	246 menopausal women with hot flashes	Promensil (82 mg of isoflavones per day) (n=84); Rimostil (57 mg of isoflavone per day) (n=83)	Placebo (less than 0.04 mg isoflavones per tablet) (n=85)	12 weeks	The change in frequency of hot flashes measured by participant daily diaries, Greene Climacteric Scale (QOL), adverse events

## Data Availability

The data used to support the findings of this study are available from the corresponding author upon request.
